# A Deletion in the *VLDLR* Gene in Eurasier Dogs with Cerebellar Hypoplasia Resembling a Dandy-Walker-Like Malformation (DWLM)

**DOI:** 10.1371/journal.pone.0108917

**Published:** 2015-02-10

**Authors:** Martina Gerber, Andrea Fischer, Vidhya Jagannathan, Michaela Drögemüller, Cord Drögemüller, Martin J. Schmidt, Filipa Bernardino, Eberhard Manz, Kaspar Matiasek, Kai Rentmeister, Tosso Leeb

**Affiliations:** 1 Institute of Genetics, Vetsuisse Faculty, University of Bern, 3001 Bern, Switzerland; 2 Section of Neurology, Clinic of Small Animal Medicine, Ludwig-Maximilians-University, 80539 Munich, Germany; 3 Department of Veterinary Clinical Science, Small Animal Clinic, Justus-Liebig-University, 35392 Giessen, Germany; 4 Generatio Sol. GmbH, 69115 Heidelberg, Germany; 5 Section of Clinical and Comparative Neuropathology, Institute of Veterinary Pathology at the Centre for Clinical Veterinary Medicine, Ludwig-Maximilians-University, 80539 Munich, Germany; 6 Tierärztliche Praxis für Neurologie, 97337 Dettelbach, Germany; University of Queensland, AUSTRALIA

## Abstract

Dandy-Walker-like malformation (DWLM) is the result of aberrant brain development and mainly characterized by cerebellar hypoplasia. DWLM affected dogs display a non-progressive cerebellar ataxia. Several DWLM cases were recently observed in the Eurasier dog breed, which strongly suggested a monogenic autosomal recessive inheritance in this breed. We performed a genome-wide association study (GWAS) with 9 cases and 11 controls and found the best association of DWLM with markers on chromosome 1. Subsequent homozygosity mapping confirmed that all 9 cases were homozygous for a shared haplotype in this region, which delineated a critical interval of 3.35 Mb. We sequenced the genome of an affected Eurasier and compared it with the Boxer reference genome and 47 control genomes of dogs from other breeds. This analysis revealed 4 private non-synonymous variants in the critical interval of the affected Eurasier. We genotyped these variants in additional dogs and found perfect association for only one of these variants, a single base deletion in the *VLDLR* gene encoding the very low density lipoprotein receptor. This variant, *VLDLR*:c.1713delC is predicted to cause a frameshift and premature stop codon (p.W572Gfs*10). Variants in the *VLDLR* gene have been shown to cause congenital cerebellar ataxia and mental retardation in human patients and *Vldlr* knockout mice also display an ataxia phenotype. Our combined genetic data together with the functional knowledge on the *VLDLR* gene from other species thus strongly suggest that *VLDLR*:c.1713delC is indeed causing DWLM in Eurasier dogs.

## Introduction

In 2005 a 199 kb deletion including the *VLDLR* gene (very low density lipoprotein receptor) was described in 8 patients of the Hutterite population with an autosomal recessive syndrome of nonprogressive cerebellar ataxia, mental retardation, inferior cerebellar hypoplasia, and mild cortical gyral simplification [1]. This study gave rise to the term “VLDLR-associated cerebellar ataxia”, which is a clinically and molecularly well-defined subgroup of the so called dysequilibrium syndrome. Dysequilibrium syndrome is a genetically heterogenous disorder, which is generally characterized by a congenital onset of autosomal recessive nonprogressive cerebellar ataxia, delayed ambulation, disturbed equilibrium and mental retardation, associated with cerebellar hypoplasia with or without quadrupedal locomotion. VLDLR associated forms of dysequilibrium syndrome were recently re-named into “cerebellar ataxia, mental retardation, and dysequilibrium syndrome 1” (CAMRQ1, OMIM #224050). So far, 13 different *VLDLR* variants were reported in human patients with dysequilibrium syndromes [1–10]. Most of the described cases descended from consanguineous parents, supporting the assumed autosomal recessive mode of inheritance.

VLDLR is a transmembrane receptor and belongs to the LDL (low density lipoprotein) receptor family. Together with the apolipoprotein E receptor 2 (APOER2), it is part of the reelin (RELN) signaling pathway controlling neuroblast migration in the cerebral cortex and cerebellum [11]. In the absence of these receptors, neuroblasts are unable to complete migration, which results in an underdeveloped central nervous system [11–13].

The *reeler* mouse mutant with a lack of functional Reln shows impaired coordination, tremors and ataxia [12]. *Vldlr* deficient mice have a smaller cerebellum than wildtype controls and show similar although less severe clinical symptoms than *reeler* mice [13]. These symptoms are similar to those of human patients with dysequilibrium syndrome.

In dogs there are several clinical and pathological reports describing cerebellar (vermian) hypoplasia and symptoms similar to human dysequilibrium syndrome [14–16]. In each of these cases the clinical signs were noted early on and may be assumed to have been present from birth. One study investigated three litters of Chow Chows and found strong evidence for an autosomal recessive inheritance [15]. To the best of our knowledge, the molecular genetic defects in the canine patients have not been published.

The Eurasier dog breed was founded in the 1960s by crossing dogs from three existing breeds, namely the Keeshound (Wolfspitz), Chow Chow, and Samoyed. As the Eurasier are a very young breed started with only a few dogs, the degree of recent inbreeding is very high with a concomitant risk of recessive inherited diseases. We recently characterized the clinical and radiological features of a cerebellar hypoplasia termed Dandy-Walker-like malformation (DWLM) in purebred Eurasier dogs [17]. The aim of the present study was to unravel the molecular genetics for this phenotype. We therefore performed a genome-wide association study (GWAS) followed by whole genome sequencing (WGS) of an affected dog to identify the disease-causing genetic variant.

## Results

### Phenotypic description and mode of inheritance

We previously described the clinical and radiological findings of DWLM in detail [17]. Briefly, the predominant clinical sign of the affected dogs in this study was a non-progressive mild to moderate cerebellar ataxia, which became evident at an early age when the dogs started to walk. Furthermore, affected dogs showed episodic rolling or leaning to one side and a subtle hypermetric gait. Some dogs also had tremors, proprioceptive deficits, nystagmus, ventral strabismus, and reduced or absent menace reflex. Behavior and learning capacity was estimated normal for breed standards. Magnetic resonance imaging (MRI) of the affected dogs revealed an absence of the caudal and middle part of the vermis and hypoplasia of the caudal parts of the cerebellar hemispheres. Some dogs showed additional supratentorial anomalies including variable degrees of hydrocephalus, thinned appearance of the corpus callosum, and incomplete septum pellucidum. We previously showed that DWLM is most likely following a monogenic autosomal recessive mode of inheritance [17].

### Mapping of the causative mutation

**Fig 1:**
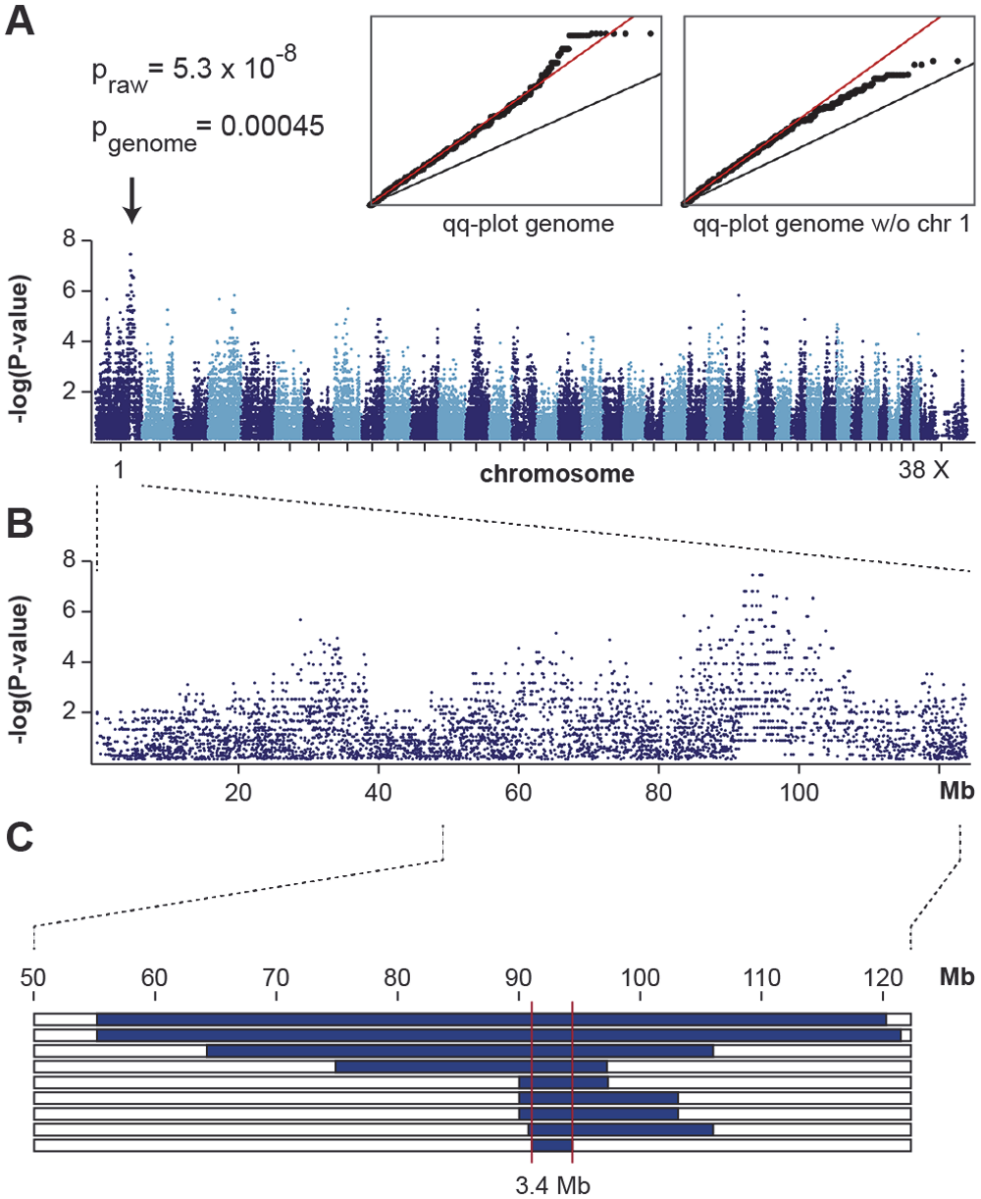
Mapping of DWLM in Eurasier dogs. (A) A genome-wide association study using 9 cases and 11 controls indicates a signal with multiple associated SNPs on chromosome 1. The p-values are inflated due to the use of closely related animals. The inserted quantile-quantile (qq) plots show the observed versus expected log p-values. The straight black line in the qq-plots indicates the distribution of SNP markers under the null hypothesis. The straight red line visualizes the inflation of p-values caused by the close relatedness. This inflation is randomly distributed across the entire genome. The skew at the right edge of the genome-wide qq-plot indicates that several markers on chromosome 1 are stronger associated with DWLM than it would be expected by chance. This skew is absent when chromosome 1 markers are omitted from the plot. (B) The detailed view of chromosome 1 suggests an associated interval of approximately 10 Mb at ~91–101 Mb. (C) Homozygosity mapping. Each horizontal bar corresponds to one of the 9 analyzed cases. Homozygous regions with shared alleles are shown in blue. A shared homozygous interval of ~3.4 Mb delineates the exact boundaries of the critical interval from 90,860,923 bp to 94,212,001 bp (CanFam 3 assembly).

We genotyped DNA samples from 9 DWLM-affected and 11 non-affected Eurasier dogs with the illumina canine_HD chip containing 173,662 evenly spaced SNPs. We pruned the raw data and removed 62,814 markers, which had low call rates or were non-informative. The final dataset thus contained 110,848 markers for the analysis. We performed a genome-wide allelic association study with this dataset. The seven best-associated SNPs were located at 92.5–93.8 Mb on chromosome 1 and had equal raw p-values of 5.3 x 10^-8^ (Fig. 1). The corrected p-value after 100,000 permutations was 0.00045.

Subsequently, we applied a homozygosity mapping approach to fine-map the region containing the causative DWLM variant. We hypothesized that the affected dogs most likely were inbred to one single founder animal. Under this scenario the affected individuals were expected to be identical by descent (IBD) for the causative mutation and flanking chromosomal segments. We analyzed the cases for extended regions of homozygosity with simultaneous allele sharing. A genome region, which coincided with the associated interval on chromosome 1, fulfilled our search criteria. Here, all 9 affected dogs were homozygous and shared identical alleles over 248 SNP markers spanning a 3.33 Mb interval. We concluded that the causative mutation should be located in the 3.35 Mb critical interval between the closest heterozygous markers on either side of the homozygous segment (chr1:90,860,923–94,212,001, CanFam 3 assembly; Fig. 1).

We additionally obtained SNP chip genotypes from 14 non-affected relatives of our DWLM cases. One obligate carrier, the clinically healthy mother of an affected dog, was also homozygous for the disease-associated alleles at 481 consecutive markers over 6.2 Mb including the entire critical interval.

### Mutation identification

A total of 39 genes and loci are annotated in the critical interval on chromosome 1 (CanFam 3.1, NCBI annotation release 103). To get a comprehensive overview of all variants in the critical interval we sequenced the whole genome of one affected Eurasier dog. We collected 144 million 2 x 100 bp read pairs from a standard 300 bp fragment library corresponding to roughly 10.6x coverage of the genome. We called SNPs and indel variants with respect to the reference genome of a non-affected Boxer. Across the entire genome, we detected 3.41 million homozygous variants (Table 1). Within the critical interval there were 150 variants, of which 20 were predicted to be non-synonymous. We further compared the genotypes of the affected Eurasier dog with 47 dog genomes of various breeds that had been sequenced in our laboratory in the course of other ongoing studies (S1 Table). We hypothesized that the mutant allele at the causative variant should be completely absent from all other dog breeds. With these filtering steps only 4 non-synonymous variants remained in the critical interval, where the affected Eurasier dog carried the homozygous variant genotype and all other 47 sequenced dogs carried the homozygous wildtype genotype (Table 2).

**Table 1:** Variants detected by whole genome re-sequencing of an affected Eurasier.

Filtering step	Number of variants
Variants in the whole genome^a^	3,412,492
Variants in the critical 3.4 Mb interval on chromosome 1	6,649
Variants in the critical interval that were absent from 47 other dog genomes	150
Non-synonymous variants in the whole genome^a^	14,211
Non-synonymous variants in the critical 3.4 Mb interval on chromosome 1	20
Non-synonymous variants in the critical interval that were absent from 47 other dog genomes	4

^a^ The sequences were compared to the reference genome (CanFam 3) from a Boxer. Only homozygous variants are reported.

**Table 2:** Four non-synonymous variants in the critical interval of an affected Eurasier that were absent from 47 other dog genomes.

Position on Chr 1 (CanFam 3 assembly)	Reference allele	Variant allele	Gene	Variant (cDNA)	Variant (protein)
91’266’144	C	-	*VLDLR*	c.1713delC	p.W572Gfs*10
93’082’389	A	C	*CDC37L1*	c.960A>C	p.L320F
93’037’453	G	A	*PPAPDC2*	c.202G>A	p.A68T
94’098’450	T	A	*SPATA31E1*	c.3782A>T	p.Q1261L

**Fig 2:**
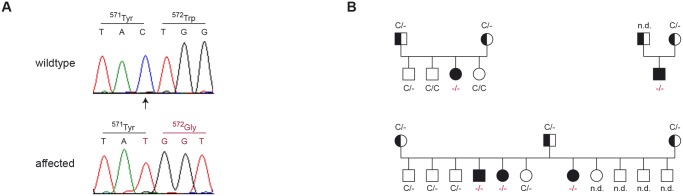
Experimental confirmation of the DWLM associated cytosine deletion by Sanger sequencing. (A) Electropherograms of the *VLDLR*:*c*.*1713delC* variant. A fragment harboring exon 12 and flanking sequences of the *VLDLR* gene was PCR-amplified and sequenced with the Sanger method. The figure shows representative traces from a normal and a DWLM affected Eurasier dog. The position of the deleted cytosine is indicated by an arrow. (B) Perfect cosegregation of the *VLDLR*:*c*.*1713delC* variant with the DWLM phenotype in four litters of Eurasier dogs. Filled symbols represent DWLM affected dogs. Obligate carriers are indicated by half-filled symbols. The transmission of the two different alleles is within the expected equal ratio and the offspring’s genotype distribution corresponds to Mendelian rules.

We confirmed all remaining non-synonymous variants by Sanger sequencing and genotyped them in larger cohorts of dogs (Table 3). Three of these variants could be excluded as being causative for DWLM, as we identified several non-affected Eurasier dogs carrying the non-reference alleles in homozygous state. Only one variant, *VLDLR*:*c*.*1713delC*, remained perfectly associated with DWLM in a cohort of 34 Eurasier dogs. This variant was absent from more than 500 dogs from other breeds. We observed perfect co-segregation of the *VLDLR*:*c*.*1713delC* variant with DWLM in two complete and two partial Eurasier litters and their parents (Fig. 2).

**Table 3:** Association of non-synonymous variants with DWLM.

Genotype	Eurasier cases (n = 9)	Eurasier obligate carriers^a^ (n = 6)	Eurasier controls (n = 19)	Dogs from other breeds^b^ (n = 546)
***VLDLR:c.1713delC***				
**C/C**	**-**	**-**	**14**	**545**
**C/del**	**-**	**6**	**5**	**-**
**del/del**	**9**	**-**	**-**	**-**
*CDC37L1:c.960A>C*				
A/A	-	-	11	47
A/C	-	5	7	-
C/C	9	1	1	-
*SPATA31E1:c.3782A>T*				
A/A	-	-	1	47
A/T	1^c^	4	13	-
T/T	8	2	5	-
*PPAPDC2:c.202G>A*				
G/G	-	-	8	46
A/G	-	1	3	-
A/A	9	3	4	-

ᵃ Parents of affected dogs were classified as obligate carriers.

^b^ These dogs consist of 47 control dogs with whole genome sequences (S1 Table) and 499 control dogs that were specifically genotyped for the *VLDLR*:*c*.*1713delC* variant (S2 Table). One of the whole genome sequences (sample BC273) did not have any coverage at the *VLDLR*:*c*.*1713delC* and the *PPAPDC2*:*c*.*202G>A* variant.

^c^ The 9 cases carried identical homozygous marker haplotypes in the critical interval. Thus the heterozygous genotype at this position in one of the cases was quite unexpected. We speculate that it may be due to an ancestral gene conversion event or, alternatively, a *de novo* revertation mutation.

The *VLDLR*:*c*.*1713delC* single nucleotide deletion results in a frameshift and premature stop codon. It is predicted to truncate more than a third of the encoded very low density lipoprotein receptor (p.W572Gfs*10).

### Carrier frequency in the Eurasier population

We genotyped a random cohort of 96 Eurasier dogs, whose DNA had been archived independently of the DWLM research study. Fifteen of these 96 dogs (16%) were heterozygous carriers of the *VLDLR*:*c*.*1713delC* variant. None of these dogs was homozygous for the variant allele.

## Discussion

Using a hypothesis-free positional cloning approach, we identified the *VLDLR*:*c*.*1713delC* variant in the canine *VLDLR* gene as the most likely causative variant for DWLM in Eurasier dogs. The single base deletion in exon 12 of the *VLDLR* gene causes a frameshift mutation, which leads to a premature stopcodon. As we had no suitable tissue samples for RNA isolation we did not assess whether the mutant transcript is subject to nonsense-mediated decay. However, even if the mutant transcript were translated, the resulting protein would lack 301 amino acids including the transmembrane domain. Thus, it seems highly likely that the mutant protein would be non-functional. We thus assume that the deletion leads to a complete loss of function.

The existing functional knowledge on the essential VLDLR function in the reelin signaling pathway and brain development [11–13] together with the similarity of the clinical symptoms of human patients with *VLDLR* mutations [1–10] very strongly support the causality of the canine variant for DWLM in Eurasier dogs.

In addition, we also would like to outline a genetic argument that virtually proves the causality of the *VLDLR*:*c*.*1713delC* variant for DWLM: In our GWAS study we included the non-affected mother of a DWLM affected dog. We confirmed the normal brain development of this dog by MRI. As this dog should represent a heterozygous carrier, we were initially quite surprised when we noticed during the analysis of the SNP chip data that it carried the disease-associated SNP haplotype in homozygous state, just like the DWLM affected dogs. The further analysis then revealed that this dog was heterozygous for the *VLDLR*:*c*.*1713delC* variant as expected for a carrier, but homozygous for every other tested variant in the critical interval. We think that the only realistic explanation for the very particular genetic makeup of this dog can be provided by a relatively recent inbreeding event to an ancestor of the DWLM founder animal. In this scenario our carrier dog must have received both copies of the disease-associated chromosome 1 haplotype from this hypothetical ancestor, which are thus virtually identical by descent. However, in one of the transmission paths this chromosome must have acquired a *de novo* deletion in the *VLDLR* gene, while the other copy of the haplotype was transmitted faithfully in its ancestral state. As *de novo* mutation events are quite rare and *VLDLR*:*c*.*1713delC* represents a clearly damaging variant in a highly plausible functional candidate gene, we think that our data prove the causality of *VLDLR*:*c*.*1713delC* beyond any reasonable doubt. The same line of genetic reasoning has previously been used to establish causality for the mutations in the ovine callipyge phenotype and one form of bovine arachnomelia [18,19].

We could not determine the founder animal of DWLM with absolute certainty. It is possible that the *VLDLR*:*c*.*1713delC* allele was already brought into the Eurasier population by one of the founding dogs. It is thus of interest that a clinically related cerebellar hypoplasia was reported in 1979 in Chow Chows [15]. Unfortunately, no DNA of these historic cases is available and it is therefore not possible to clarify whether they were due to the same genetic defect. As our results will enable genetic testing we recommend that the absence of this disease allele should be experimentally verified in a representative cohort of Chow Chows.

In conclusion we identified a single base deletion in the *VLDLR* gene in dogs with cerebellar hypoplasia and ataxia, a phenotype termed Dandy-Walker-like malformation (DWLM). Our results enable genetic testing and the eradication of this inherited disease in the Eurasier dog population and provide a defined animal model to develop a better understanding for VLDLR associated cerebellar hypoplasia and dementia in humans.

## Materials and Methods

### Ethics statement

All animal experiments were performed according to the local regulations. The dogs in this study were examined with the consent of their owners. The study was approved by the “Cantonal Committee For Animal Experiments” (Canton of Bern; permit 23/10).

### Animal selection

We obtained EDTA blood samples from 9 affected and 25 non-affected Eurasier dogs. The phenotypes were based on clinical neurological examination and MRI imaging as described [17]. Four of the cases were full-siblings. The complete cohort for this study consisted of 34 Eurasier dogs and 546 dogs of diverse other breeds (S1 Table, S2 Table). For all of these samples a non-affected phenotype was assumed (“population controls”) as the investigated cerebellar hypoplasia is a young and relatively rare trait.

An additional cohort of 96 Eurasier dogs of the Kynologische Zuchtgemeinschaft Eurasier e.V. was used to determine the carrier frequency. Blood samples of these dogs had been submitted to the diagnostic lab Generatio Sol. on behalf of the owners.

### DNA samples and SNP genotyping

We isolated genomic DNA samples from EDTA blood with the Nucleon Bacc2 kit (GE Healthcare). Genotyping on illumina canine_HD chips containing 173,662 SNP markers was performed by GeneSeek. Genotypes were stored in a BC/Gene database version 3.5 (BC/Platforms).

### Genome-wide association study (GWAS) and homozygosity mapping

We used PLINK v1.07 [20] to perform genome-wide association analyses (GWAS). We removed markers and individuals with call rates < 90% from the analysis. We also removed markers with minor allele frequency (MAF) < 5% and markers strongly deviating from Hardy-Weinberg equilibrium (p < 10^-5^). We performed an allelic association study and determined an empirical significance threshold by performing 100,000 permutations of the dataset with arbitrarily assigned phenotypes. It has to be cautioned that the samples were highly stratified and the genomic inflation factor in this analysis was 2.57. This extremely high value was probably caused by the use of closely related dogs including two full-sib pairs among the cases. When we tried to correct for the stratification by using the GenABEL software and a mixed-model analysis [21] we did no longer see any genome-wide significant association (data not shown). We used PLINK to search for extended intervals of homozygosity with shared alleles.

### Gene analysis

We used the dog CanFam 3 genome assembly derived from a Boxer genome. All numbering within the canine *VLDLR* gene corresponds to the accessions NM_001286978 (mRNA) and NP_001273907 (protein).

### Whole genome sequencing of an affected Eurasier dog

We prepared a fragment library with 300 bp insert size and collected one lane of illumina HiSeq2500 paired-end reads (2 x 100 bp). We obtained a total of 287,996,124 paired-end reads or roughly 10x coverage. We used fastq-mcf to clean the raw dataset (http://code.google.com/p/ea-utils). Fastq-mcf found 50,191,228 reads too short (less than 50 bases) after clipping for bad quality bases and Ns from the 3’-end. We mapped the reads to the dog reference genome using the Burrows-Wheeler Aligner (BWA) version 0.5.9-r16 [22] with default settings. After sorting the mapped reads by the coordinates of the sequence with Picard tools, we labeled 1.4% of the reads as PCR and optical duplicates also with Picard tools (http://sourceforge.net/projects/picard/). We used the Genome Analysis Tool Kit (GATK version 0591, [23]) to perform local realignment and to produce a cleaned BAM file. In the clean BAM file we had 229,719,638 mapped reads and 225,831,256 (95%) uniquely mapping reads. Variants calls were then made with the unified genotyper module of GATK. Variant data for each sample were obtained in variant call format (version 4.0) as raw calls for all samples and sites flagged using the variant filtration module of GATK. Variant calls that failed to pass the following filters were labeled accordingly in the call set: (i) Hard to Validate MQ0 ≥ 4 & ((MQ0 / (1.0 * DP)) > 0.1); (ii) strand bias (low Quality scores) QUAL < 30.0 || (Quality by depth) QD < 5.0 || (homopolymer runs) HRun > 5 || (strand bias) SB > 0.00; (iii) SNP cluster window size 10. The snpEFF software [24] together with the CanFam 3.1 annotation was used to predict the functional effects of detected variants.

### Sanger sequencing

We used Sanger sequencing to confirm the illumina sequencing results and to perform targeted genotyping for selected variants. For these experiments we amplified PCR products using AmpliTaqGold360Mastermix (Applied Biosystems). PCR products were directly sequenced on an ABI 3730 capillary sequencer (Applied Biosystems) after treatment with exonuclease I and shrimp alkaline phosphatase. We analyzed the sequence data with Sequencher 5.1 (GeneCodes).

## Supporting Information

S1 Table Dogs used for whole genome sequencing. (XLSX)

S2 Table Control dogs from other breeds. (XLSX)

## References

[1] Boycott KM, Flavelle S, Bureau A, Glass HC, Fujiwara TM, et al (2005) Homozygous deletion of the very low density lipoprotein receptor gene causes autosomal recessive cerebellar hypoplasia with cerebral gyral simplification. Am J Hum Genet 77: 477–483. 16080122 10.1086/444400 PMC1226212

[2] Moheb LA, Tzschach A, Garshasbi M, Kahrizi K, Darvish H, et al (2008) Identification of a nonsense mutation in the very low-density lipoprotein receptor gene (VLDLR) in an Iranian family with dysequilibrium syndrome. Eur J Hum Genet 16: 270–273. 18043714 10.1038/sj.ejhg.5201967

[3] Sonmez FM, Gleeson JG, Celep F, Kul S (2013) The very low density lipoprotein receptor-associated pontocerebellar hypoplasia and dysmorphic features in three Turkish patients. J Child Neurol 28: 379–383. 10.1177/0883073812441065 22532556 PMC4442636

[4] Ozcelik T, Akarsu N, Uz E, Caglayan S, Gulsuner S, et al (2008) Mutations in the very low-density lipoprotein receptor VLDLR cause cerebellar hypoplasia and quadrupedal locomotion in humans. Proc Natl Acad Sci 105: 4232–4236. 10.1073/pnas.0710010105 18326629 PMC2393756

[5] Türkmen S, Hoffmann K, Demirhan O, Aruoba D, Humphrey N, et al (2008) Cerebellar hypoplasia, with quadrupedal locomotion, caused by mutations in the very low-density lipoprotein receptor gene. Eur J Hum Genet 16: 1070–1074. 10.1038/ejhg.2008.73 18364738

[6] Kolb LE, Arlier Z, Yalcinkaya C, Ozturk AK, Moliterno JA, et al (2010) Novel VLDLR microdeletion identified in two Turkish siblings with pachygyria and pontocerebellar atrophy. Neurogenetics 11: 319–325. 10.1007/s10048-009-0232-y 20082205

[7] Boycott KM, Bonnemann C, Herz J, Neuert S, Beaulieu C, et al (2009) Mutations in VLDLR as a cause for autosomal recessive cerebellar ataxia with mental retardation (dysequilibrium syndrome). J Child Neurol 24: 1310–1315. 10.1177/0883073809332696 19332571 PMC2849979

[8] Ali BR, Silhavy JL, Gleeson MJ, Gleeson JG, Al-Gazali L (2012) A missense founder mutation in VLDLR is associated with dysequilibrium syndrome without quadrupedal locomotion. BMC Med Genet 13: 80 10.1186/1471-2350-13-80 22973972 PMC3495048

[9] Schlotawa L, Hotz A, Zeschnigk C, Hartmann B, Gärtner J, et al (2013) Cerebellar ataxia, mental retardation and dysequilibrium syndrome 1 (CAMRQ1) caused by an unusual constellation of VLDLR mutation. J Neurol 260: 1678–1680. 10.1007/s00415-013-6941-z 23670308

[10] Dixon-Salazar TJ, Silhavy JL, Udpa N, Schroth J, Bielas S, et al (2012) Exome sequencing can improve diagnosis and alter patient management. Sci Transl Med 4: 138ra78 10.1126/scitranslmed.3003544 22700954 PMC4442637

[11] Förster E, Bock HH, Herz J, Chai X, Frotscher M, et al (2010) Emerging topics in reelin function. Eur J Neurosci 31: 1511–1518. 10.1111/j.1460-9568.2010.07222.x 20525064 PMC2942760

[12] D’Arcangelo G, Miao GG, Chen SC, Soares HD, Morgan JI, et al (1995) A protein related to extracellular matrix proteins deleted in the mouse mutant reeler. Nature 374: 719–723. 7715726 10.1038/374719a0

[13] Trommsdorff M, Gotthardt M, Hiesberger T, Shelton J, Stockinger W, et al (1999) Reeler/Disabled-like disruption of neuronal migration in knockout mice lacking the VLDL receptor and ApoE receptor 2. Cell 97: 689–701. 10380922 10.1016/s0092-8674(00)80782-5

[14] Kornegay JN (1986) Cerebellar vermian hypoplasia in dogs. Vet Pathol 23: 374–379. 3750731 10.1177/030098588602300405

[15] Knecht CD, Lamar CH, Schaible R, Pflum K (1979) Cerebellar hypoplasia in Chow Chows. J Am Anim Hosp Assoc 15: 51–53.

[16] Schmid V, Lang J, Wolf M (1992) Dandy-Walker like syndrome in four dogs: cisternography as a diagnostic aid. J Am Anim Hosp Assoc 28: 355–360.

[17] Bernardino F, Rentmeister K, Schmidt MJ, Brühschwein A, Matiasek K, et al (2015) Cerebellar hypoplasia resembling Dandy-Walker-like malformation in purebed Eurasier dogs with familial non-progressive ataxia: a retrospective and prospective clinical cohort study. PLoS ONE 10(2): e0117670 25668516 10.1371/journal.pone.0117670 PMC4323131

[18] Freking BA, Murphy SK, Wylie AA, Rhodes SJ, Keele JW, et al (2002) Identification of the single base change causing the callipyge muscle hypertrophy phenotype, the only known example of polar overdominance in mammals. Genome Res 12: 1496–1506. 12368241 10.1101/gr.571002 PMC187527

[19] Drögemüller C, Tetens J, Sigurdsson S, Gentile A, Testoni S, et al (2010) Identification of the bovine arachnomelia mutation by massively parallel sequencing implicates sulfite oxidase (SUOX) in bone development. PLOS Genetics 6: e1001079 10.1371/journal.pgen.1001079 20865119 PMC2928811

[20] Purcell S, Neale B, Todd-Brown K, Thomas L, Ferreira MA, et al (2007) PLINK: a tool set for whole-genome association and population-based linkage analyses. Am J Hum Genet 81: 559–575. 17701901 10.1086/519795 PMC1950838

[21] Aulchenko YS, Ripke S, Isaacs A, van Duijn C (2007) GenABEL: an R library for genome-wide association analysis. Bioinformatics, 23: 1294–1296. 17384015 10.1093/bioinformatics/btm108

[22] Li H, Durbin R (2009) Fast and accurate short read alignment with Burrows-Wheeler transform. Bioinformatics 25: 1754–1760. 10.1093/bioinformatics/btp324 19451168 PMC2705234

[23] McKenna A, Hanna M, Banks E, Sivachenko A, Cibulskis K, et al (2010). The genome analysis toolkit: a MapReduce framework for analyzing next-generation DNA sequencing data. Genome Res 20: 1297–1303. 10.1101/gr.107524.110 20644199 PMC2928508

[24] Cingolani P, Platts A, Coon M, Nguyen T, Wang L, et al (2012) A program for annotating and predicting the effects of single nucleotide polymorphisms, SnpEff: SNPs in the genome of Drosophila melanogaster strain w1118; iso-2; iso-3. Fly 6: 80–92. 10.4161/fly.19695 22728672 PMC3679285

